# A systematic review of the relationship between normal range of serum thyroid-stimulating hormone and bone mineral density in the postmenopausal women

**DOI:** 10.1186/s12905-023-02488-9

**Published:** 2023-07-05

**Authors:** Xiaoli Zhu, Man Li, Xinying Dong, Fen Liu, Shugang Li, Yifei Hu

**Affiliations:** 1 Department of Child, Adolescent Health and Maternal Care, School of Public Health, Capital Medical University, No. 10 You’ anmenwai Xitoutiao, Fengtai District, Beijing, 100069 China; 2 Health Service Department of the Guard Bureau of the Joint Staff Department, Beijing, China

**Keywords:** Serum thyroid-stimulating hormone, Bone mineral density, Osteoporosis, Postmenopausal women

## Abstract

**Objective:**

The aim of this study was adopts meta-analysis in evaluating the correlation between TSH and BMD, as well as osteoporosis in the postmenopausal women with normal thyroid function.

**Methods:**

Six databases were searched for articles concerning correlation between TSH and BMD in postmenopausal women. The retrieval time was set from the date of database establishment to November 30, 2020. Revman5.3 and Stata12.0 software were used for meta-analysis.

**Results:**

A total of 19 articles were incorporated. The Summary Fisher’ Z of the correlation between TSH and BMD was 0.16, 95% CI (0.00, 0.32), and the correlation coefficient of Summary Fisher’ Z conversion was 0.158. Study on the relationship between TSH and osteoporosis based on OR demonstrated that the combined OR was 1.76, 95% CI (1.27, 2.45), P < 0.05. The subgroup analyzing results displayed that the risk of osteoporosis of the subjects from community with low TSH was 1.89, 95% CI (1.43, 2.49). The risk of osteoporosis for subjects with low TSH and from hospitals was 1.36, 95% CI (0.46, 3.99); 1.84 for subjects with low TSH and anti-osteoporosis drugs, 95% CI (1.05, 3.22); and 1.74 for those with low TSH but not taking anti-osteoporosis drugs, 95% CI (1.08, 2.82). The dose-response relationship showed that the risk of osteoporosis tended to decrease when TSH was more than 2.5mIu/L.

**Conclusion:**

The serum TSH is positively related with BMD in postmenopausal women, and high TSH (> 2.5 mIu/L) within the normal range is possibly helpful to decrease the risk of osteoporosis in postmenopausal women.

**Supplementary Information:**

The online version contains supplementary material available at 10.1186/s12905-023-02488-9.

Osteoporosis(OP) is a condition resulting in an increased risk of skeletal fractures due to a reduction in the density of bone tissue [1]. and it is common in postmenopausal women. About 30%of postmenopausal women in Asian countries have OP [2]. It is believed that OP serves as the biggest trigger for osteoporotic fracture, and it can elevate the occurrence of fractures, for every 10% decrease in bone mineral density (BMD), the risk of fracture will increase by 2–3 times [3]. Research findings that more than 15% of postmenopausal women may have a hip fracture, and 50% of postmenopausal women may have a lifelong osteoporosis fracture [4]. Therefore, know about the influencing factors of OP in postmenopausal women are of great significance to the public health.

The occurrence of OP is related to gender, age, hormones, etc. [5]. Among them, hormones are believed to be closely related to OP, and thus gained great attention. In postmenopausal women, the decreased in estrogen levels also leads to changes in other hormones, which can significantly increase the incidence of OP [6]. Previous studies have illustrated that the occurrence of OP is related to thyroid hormone. Abe et al. [7] performed the experiments on the TSHR knockout mouse model and found that the TSH level of the TSHR knockout homozygous mice increased, but the BMD level decreased. Exogenous thyroid hormone supplementation did not reverse bone mineral density loss, suggesting that thyroid stimulating hormone (TSH) may be an independent risk factor for OP.

However, we found that there are a lot of controversies about the relationship between serum TSH and BMD of postmenopausal women, based on the published articles or evidence. Cui Xinjie et al. [8] found that serum TSH and lumbar BMD in postmenopausal women with normal thyroid function was positively correlated. However it was reported that serum TSH in postmenopausal women is negatively correlated with lumbar vertebral BMD by Wang Yi [9]. Besides, Yin Fei et al. [10].did not discover the statistical correlation between the serum TSH and the total hip BMD of postmenopausal. There is still no meta-analysis on the exact relationship between serum TSH levels and OP in postmenopausal women with normal thyroid function. Thus, the present systematic review attempted to identify the Relationship between Normal Range of serum thyroid-stimulating hormone and bone mineral density in the postmenopausal women.

## Materials and methods

### Articles searching strategy

**Fig. 1:**
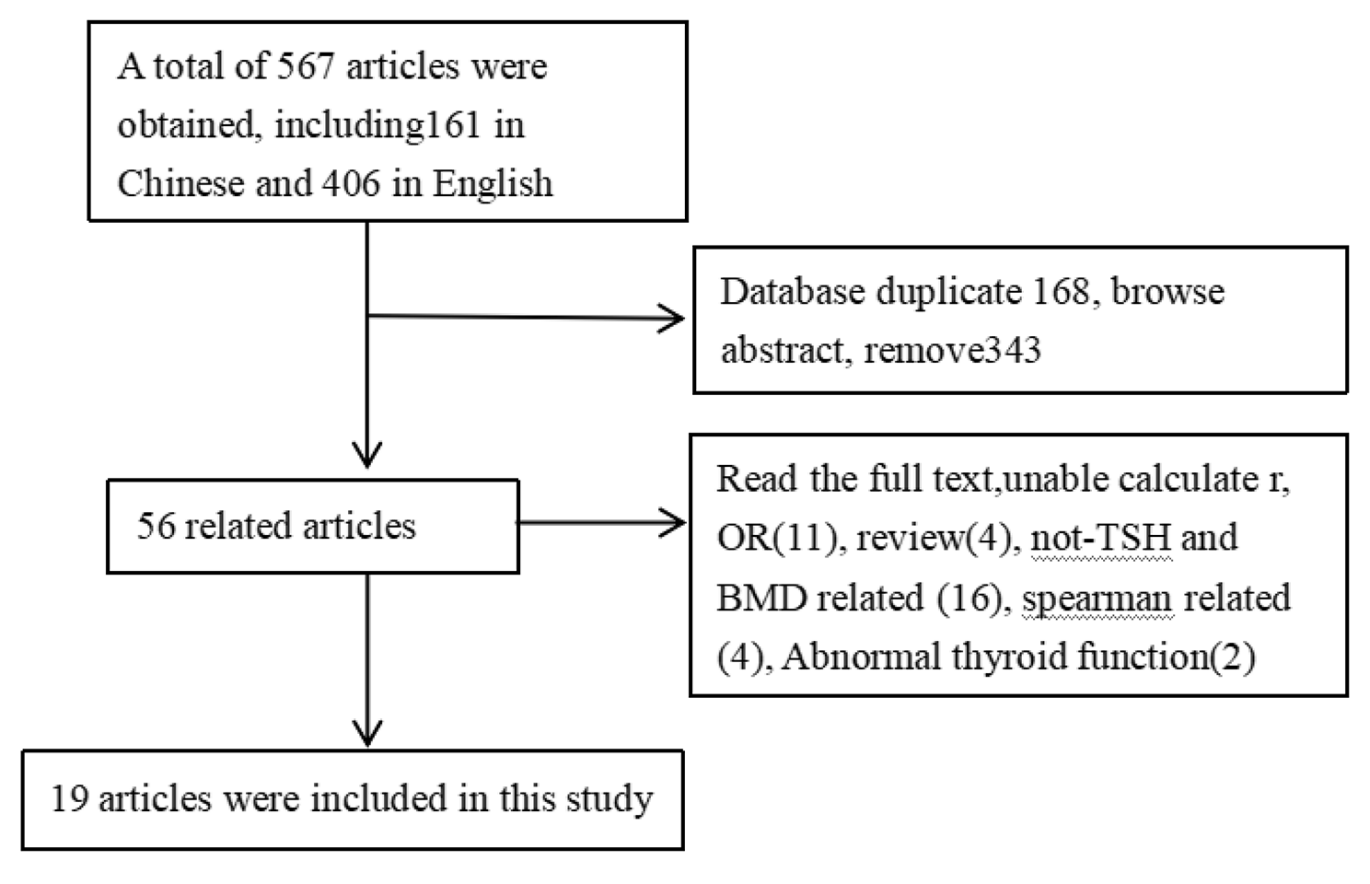
Search process and results

In this study, a total 6 databases were retrieved, we are proficient in English and Chinese, so we searched articles in English in Cochrane Library, PubMed and Web of Science. For Chinese, we searched in CNKI, Wanfang and VIP, and the retrieval time of each database was from database construction to November 30, 2020. See details in Fig. 1.

The key words mainly included the following: postmenopausal women, older women, thyroid-stimulating hormone (TSH), BMD, osteoporosis. Taking Pubmed as an example, the specific retrieval formula is as follows: ((((Postmenopausal women) OR Older women)) AND ((thyroid-stimulating hormone) OR TSH)) AND ((BMD) OR Osteoporosis). When the same article is published in both Chinese and English, we choose the English article for analysis and select according to the inclusion and exclusion criteria.

### Inclusion criteria

① Observational studies in both Chinese and English version; ② Research variables are TSH and OP; ③ Outcome index is the correlation relationship and the effective index is r, OR, mean; ④ The research objects in the original articles are postmenopausal women; ⑤ Articles are either in Chinese or English.

### Exclusion criteria

① Review articles; ② Spearman correlation coefficient articles; ③ Studies with incomplete data or offer no way to extract the calculated r, OR; ④ Articles unrelated to correlation coefficient between TSH and BMD; ⑤ Abnormal thyroid function.

### Statistical analyses

The correlation coefficient of less than 0.5 does not observe the normal distribution. As it moves to more than 0.5, the result remains the same, hence Fisher proposed to use the “Fisher’ Z transformation” formula for conversion, which adapts the correlation coefficient r into a normally distributed variable Z. Since this study adopted meta-analysis based on the Pearson’s correlation coefficient, the “Fisher’ Z transformation” [11] formula could be used for conversion. The specific conversion formula is as follows:

1 $$Fisher{^{\prime}}\,\,Z{\rm{ }} = {\rm{ 0}}{\rm{.5}} \times {{1 + r} \over {1 - r}}$$

2 $$SE\, = \,\sqrt {\frac{1}{{n - 1}}}$$

3 $$Summary{\rm{ }} = {{{e^{2Z}} - 1} \over {{e^{2Z}} + 1}}(Z{\rm{ }}is{\rm{ }}summary{\rm{ }}Fisher{^{\prime}}\,\,Z{\rm{ }}value)$$

Our study applied Revman5.3 software for statistical analysis, and P < 0.05 was considered statistically significant. EXCEL2010 and Stata12.0 software was used to calculate converted data, draw a dose-response diagram. The above-mentioned formula was adopted to convert the data taking correlation coefficient r as the outcome variable, and thus the Fisher’ Z and standard error (SE) were obtained. Secondly, the Revman5.3 software was applied to perform the inverse variance method, to obtain the summary Fisher’ Z value. Finally, we adopted the formula ③ to gain the combined effect summary r of the correlation coefficient. All of these steps were taken to evaluate the correlation between serum TSH and BMD. In terms of the studies on the relationship between TSH and the risk of OP, most of the articles included only reported the effect size and its 95% CI, and most of them were adjusted for confounding factors. Therefore, the Revman5.3 software can calculate SE and the logarithm of OR (log OR) and then its combined effects observed. Two independent reviewers performed the data extraction, and a third reviewer was consulted for any uncertainties.

### Risk of Bias across Studies

#### Publication Bias

We used a funnel plot and Egger’ s test to evaluate whether there was a publication bias in the included articles.

#### Sensitivity analysis

The Stata12.0 software was applied for sensitivity analysis. The Chi-square test was performed withα = 0.05 as the significance level; when P < 0.05, the difference was considered statistically significant.

We mainly used the Review Manger 5.3 and Stata12.0 software for data analysis. Heterogeneity is divided into two degrees according to I^2^, I^2^ < 50% is low heterogeneity that is acceptable, I^2^ ≥ 50% is high heterogeneity, andα = 0.05 was applied as the significance level for hypothesis testing of heterogeneity I^2^. When P < 0.05, I^2^ ≥ 50%, indicating heterogeneity among multiple studies, the combined effect of OR and its 95% confidence interval is estimated by the random-effects models, and when P > 0.05, I2 < 50%, indicating homogeneity among multiple studies, the fixed-effect model was used to estimate the combined effect and its 95% confidence interval. When the heterogeneity is high, subgroup analysis will be conducted according to the source of the study objects, the detection site of bone mineral density, and the use of anti-osteoporosis drugs in order to find the source of heterogeneity.

**Table 1:** Basic characteristics of the included articles

Serial number	Publication year	Author	Country	Language	Main outcome indicators
1	2006	Duk Jae kim [12]	Korea	English	OR
2	2007	Martha. Savaria Morris [13]	American	English	OR
3	2010	Gherardo Mazziotti [14]	Italy	English	OR
4	2011	Junn-Diann Lin [15]	China	English	r
5	2014	Avi Leader [16]	Israel	English	OR
6	2015	H._M.Noh [17]	Korea	English	OR
7	2016	Yin Fei (Chinese) [10]	China	Chinese	r, mean
8	2016	Berrin Acar [18]	Turkey	English	OR
9	2016	Lin Mei (Chinese) [19]	China	Chinese	r
10	2016	Bo Ding [20]	China	English	OR, mean
11	2016	SuJinLee [21]	Korea	English	OR, mean
12	2017	Wang Jiadan (Chinese) [22]	China	Chinese	R, mean
13	2018	Niu Fengxiu (Chinese) [23]	China	Chinese	r
14	2018	Qin Liping (Chinese) [24]	China	Chinese	OR
15	2018	Wang Yi (Chinese) [9]	China	Chinese	R, mean
16	2019	Gao Saisai (Chinese) [25]	China	Chinese	R, mean
17	2019	Zhang Lihong (Chinese) [26]	China	Chinese	r
18	2019	Chen Qingling(Chinese) [27]	China	Chinese	OR
19	2020	Cui Xinjie (Chinese) [8]	China	Chinese	r

The method recommended by the Agency for Health care Research and Quality (AHRQ) is adopted in evaluating the quality of the cross-sectional studies. It contains 11 items, with a maximum score of 11 points. Articles scored 0–3 points, 4–7 points, and 8–11 points are classified into low quality, medium quality, and high-quality respectively (Additional file 3).19 articles were included in this study are of medium quality.

The quality assessment was independently conducted by the first author, and the second author checked and collated the results in detail. Discussed and resolved any disagreement with the third author.

## Results

### Basic information of the included articles

This study included 19 articles with 23,960 subjects, and the publication time ranged from 2006 to 2020. Among which, 12 articles were published in China, 3 in South Korea, 1 in the United States, Italy, Israel, and Turkey respectively (Table 1).

### The relationship between serum TSH and BMD in postmenopausal women based on the Pearson correlation coefficient

The effect sizes were all Pearson correlation coefficients. The value of the correlation coefficient r in the previous studies was converted by the above-mentioned formula, and shown in Table 2.

**Fig. 2:**
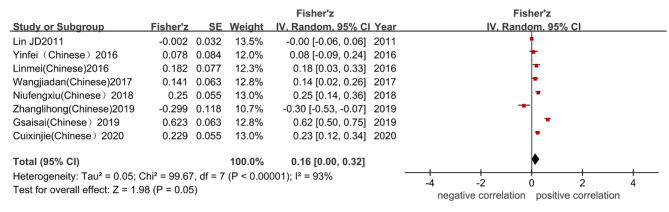
Meta-analysis of correlation between serum TSH and BMD.

We found that TSH was positively correlated with BMD, Fisher’ Z = 0.16, 95% CI (0.00, 0.32), Z = 1.98, P = 0.05 (Fig. 2). The final combined effect value r of TSH and BMD was 0.158.

**Table 2:** Analytical values obtained from data conversion

Year	author	sample	r	Fisher’ Z	SE	95%CI
2011	Lin JD [15]	974	-0.002	-0.002	0.032	(-0.06,0.06)
2016	Yin Fei (Chinese) [10]	135	0.078	0.078	0.084	(-0.09,0.24)
2016	Lin Mei (Chinese) [19]	166	0.180	0.182	0.077	(0.03,0.33)
2017	Wang Jiadan (Chinese) [22]	234	0.140	0.141	0.063	(0.02,0.26)
2018	Wang Yi (Chinese) [9]	110	-0.991	-2.649	0.095	(-2.84,-2.46)
2018	Niu Fengxiu (Chinese) [23]	308	0.245	0.250	0.055	(0.14,0.36)
2019	Gao Saisai (Chinese) [25]	267	0.535	0.623	0.063	(0.50,0.75)
2019	Zhang Lihong (Chinese) [26]	72	-0.290	-0.299	0.118	(-0.53, -0.07)
2020	Cui Xinjie (Chinese) [8]	307	0.225	0.229	0.055	(0.12,0.34)

**Fig. 3:**
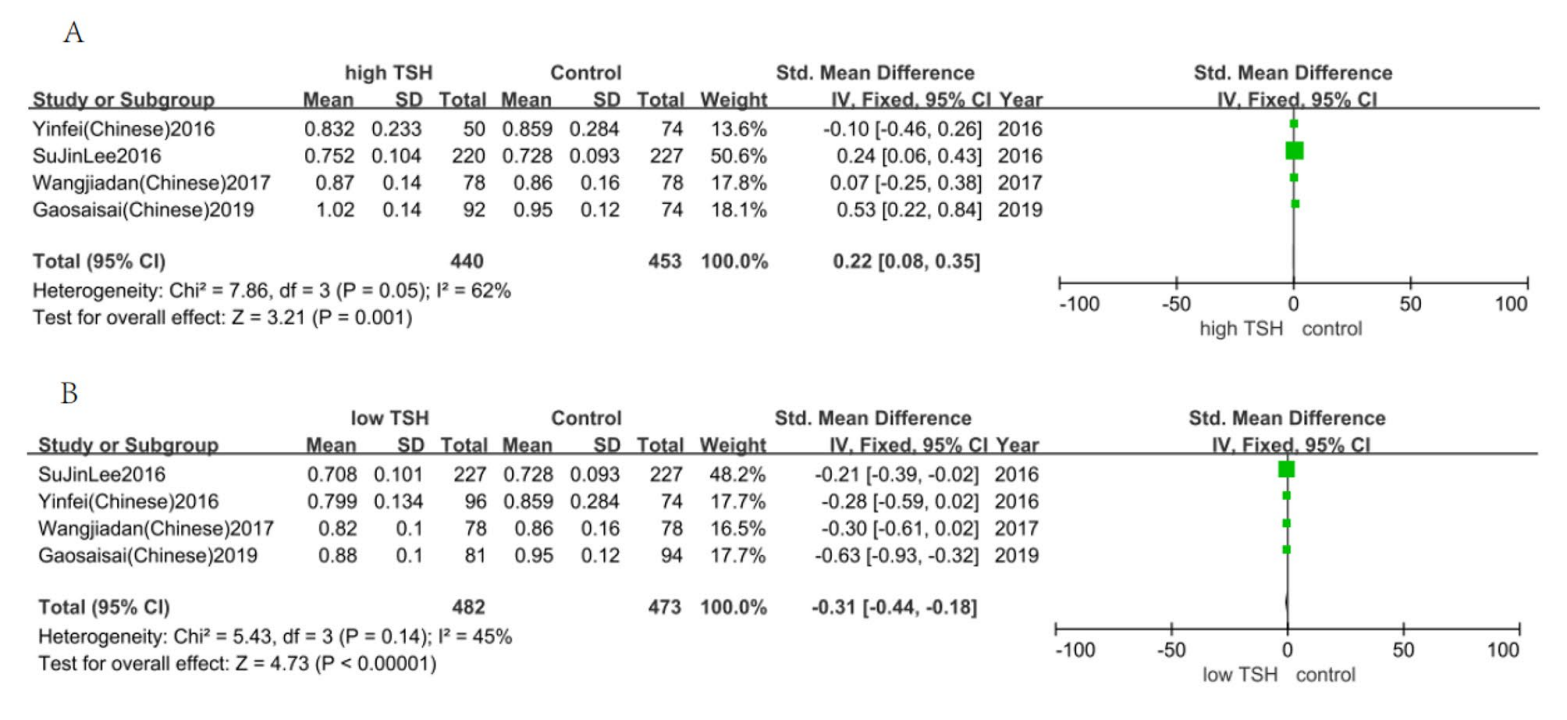
Comparison of BMD between high-level/low-level TSH group and control group

### BMD comparison among TSH groups with different levels in postmenopausal women with normal thyroid function

In original studies, the TSH was trisected according to the Tri -sectional quantiles, namely low, medium, and high. The cutoff value was incorporated into the previous group. We took the middle-level TSH group was used as the control in this study, to analyze the difference in BMD between the high-level TSH group and the low-level TSH group. The BMD of the high-level TSH group was higher than that of the control group, with SMD of 0.22, 95% CI (0.08, 0.35), P = 0.001. The BMD of the low-level TSH group was statistically lower than the control group, SMD at -0.31, 95% CI (-0.44, -0.18), P < 0.001 (Fig. 3).

### The relationship between TSH and osteoporosis

Multivariate logistic regression could determine the frequency of osteoporosis in different groups with different TSH levels after adjusting confounding factors (age, BMI, BMD, usage of anti-osteoporosis drugs), combine the effect size OR included in the article.

**Fig. 4:**
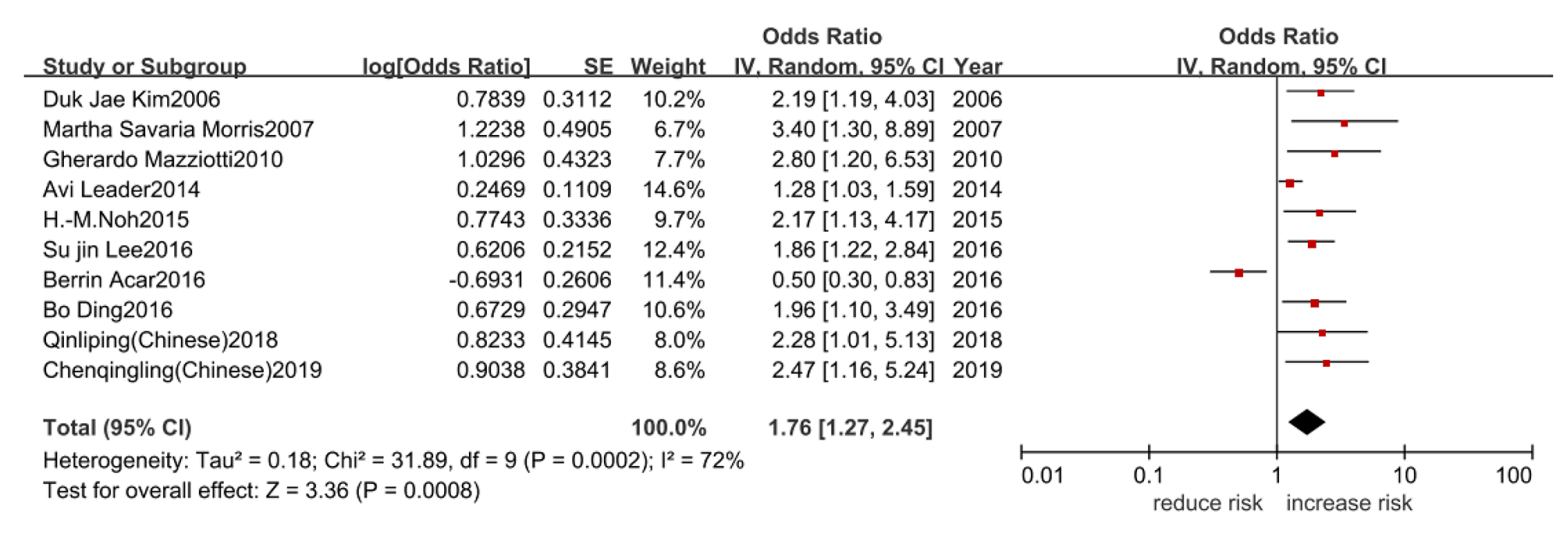
Meta-analysis of correlation between serum TSH and OP.

We found that the risk of osteoporosis for low level TSH was 1.76 times, 95% CI (1.27, 2.45) of that high level TSH (Fig. 4). This indicates that low level TSH will increase the dependence of OP.

### Results of subgroup analysis

A subgroup analysis of the source-based research subjects with OR as the measurement index, found that low level TSH group in the community facing higher risk of osteoporosis, OR = 1.89, 95%CI (1.43, 2.49), P < 0.01. In addition, whether anti-osteoporosis drugs were taken or not, low level TSH increased the risk of osteoporosis, OR at [1.84, 95%CI (1.05, 3.22), P = 0.03] and [1.74, 95%CI (1.08, 2.82), P = 0.02] respectively. The results of subgroup analysis of studies with the Pearson correlation coefficient as the outcome index could be found from Table 3. The subjects took calcium and other anti-osteoporosis drugs, summary Fisher’ Z = 0.14, 95%CI (0.02, 0.26), P = 0.03, which could affect the relationship between TSH and BMD. BMD detection site, whether the subjects suffered diabetes or not etc, had no influence on the relationship between TSH and BMD (Table 4).

**Table 3:** Subgroup analysis to determine the influencing factors of the relationship between TSH level and OP

Grouping factors	Grouping standard	Number of articles	OR 95%CI	I^2^	P
**Source of research objects**	community	7	1.89 (1.43,2.49)	44%	< 0.01
	hospital	3	1.36 (0.46,3.99)	89%	0.58
**Use of anti-OP medications**	taking anti-OP drugs	3	1.84 (1.05,3.22)	65%	0.03
	not taking anti-OP drugs	7	1.74 (1.08, 2.82)	77%	0.02
total		10	1.76 (1.27,2.45)	72%	< 0.01

**Table 4:** Subgroup analysis to determine the influencing factors of the relationship between TSH level and BMD

Grouping factors	Grouping standard	Number of articles	Summary Fisher’ Z 95%CI	I^2^	P
**Detection site**	Hip	3	0.28 (-0.07,0.63)	95%	0.11
	Lumbar spine	4	0.12 (-0.06,0.29)	84%	0.18
	Wrist	1	-0.00 (-0.06,0.06)	-	0.95
**Use of anti-OP medications**	taking anti-OP drugs	1	0.14 (0.02,0.26)	-	0.03
	not taking anti-OP drugs	7	0.16 (-0.02,0.34)	94%	0.08
**Has diabetes**	yes	5	0.19 (-0.05,0.43)	93%	0.11
	no	2	0.06 (-0.08,0.20)	76%	0.40
	Not reported	1	0.18 (0.03, 0.33)	-	0.02
Total		8	0.16 (0.00,0.32)	93%	

### The dose-response relationship between different TSH levels and osteoporosis

It could be seen in additional file 4 that OR of different TSH levels and OP, including 5 research [12, 16, 17, 21, 27] to reflect the dose-response relationship, each study used the high-level group as a control to analyzed the risk of osteoporosis in the different levels of TSH group within the normal range. The TSH level in the table was the average level. We employed Stata12.0 software to draw a dose-response diagram.

**Fig. 5:**
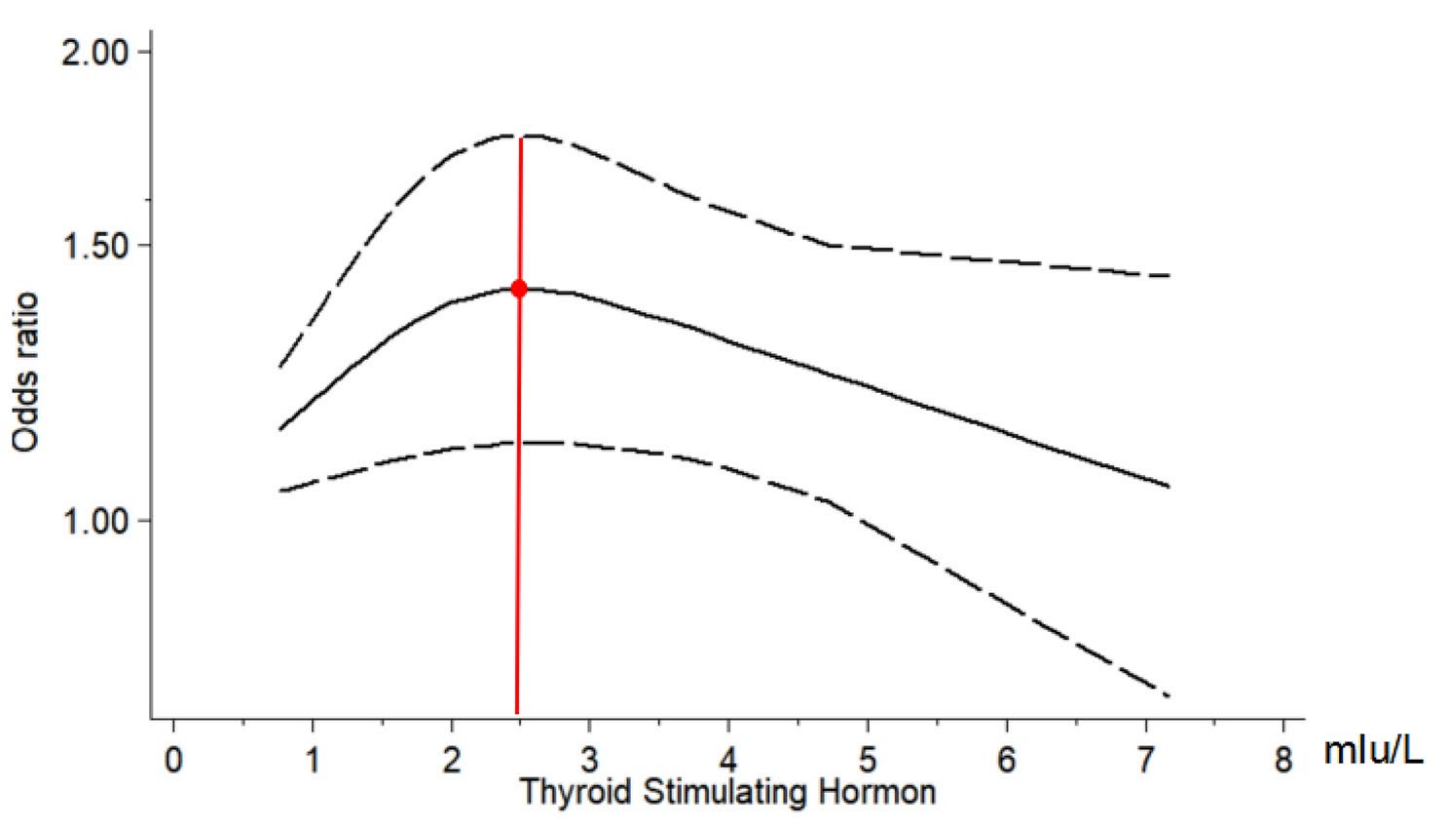
The dose-response relationship of osteoporosis at different TSH levels

According to the dose-response relationship, we knew that even when TSH was within the normal range, TSH = 2.5mIU/L, the risk of osteoporosis kept in a high level. When it was lower or higher than 2.5mIU/L, the development of OP could be diminished, and the risk of osteoporosis gradually decreased with the increasing TSH level (Fig. 5).

### Sensitivity analysis

**Fig. 6:**
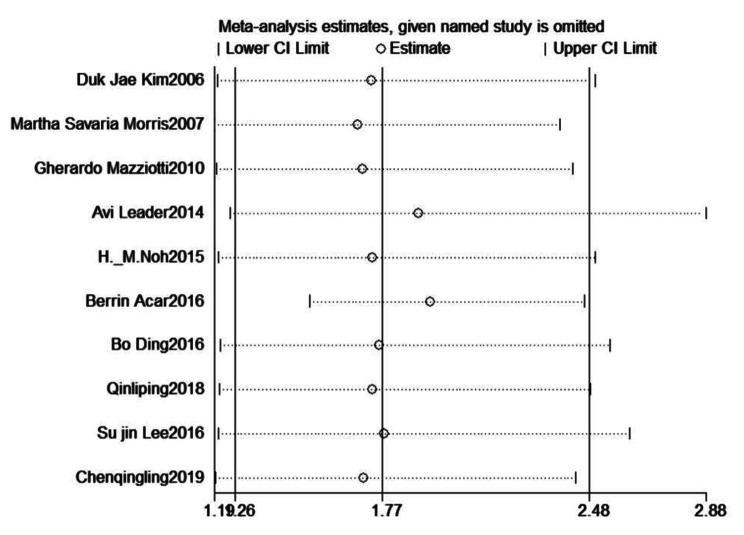
Sensitivity analysis results based on OR value

After eliminating included articles one by one, it was found that with Wang Yi [9] Summary Fisher’ Z value at -2.65, and 95% CI (-2.84, -2.46), the conclusion of the study was totally opposite. Therefore, this article was excluded in the subsequent analysis (Additional file 5). In the research of the relationship between TSH and osteoporosis based on the OR value, the results were relatively stable after screening each studies (Fig. 6).

### Publication bias

**Fig. 7:**
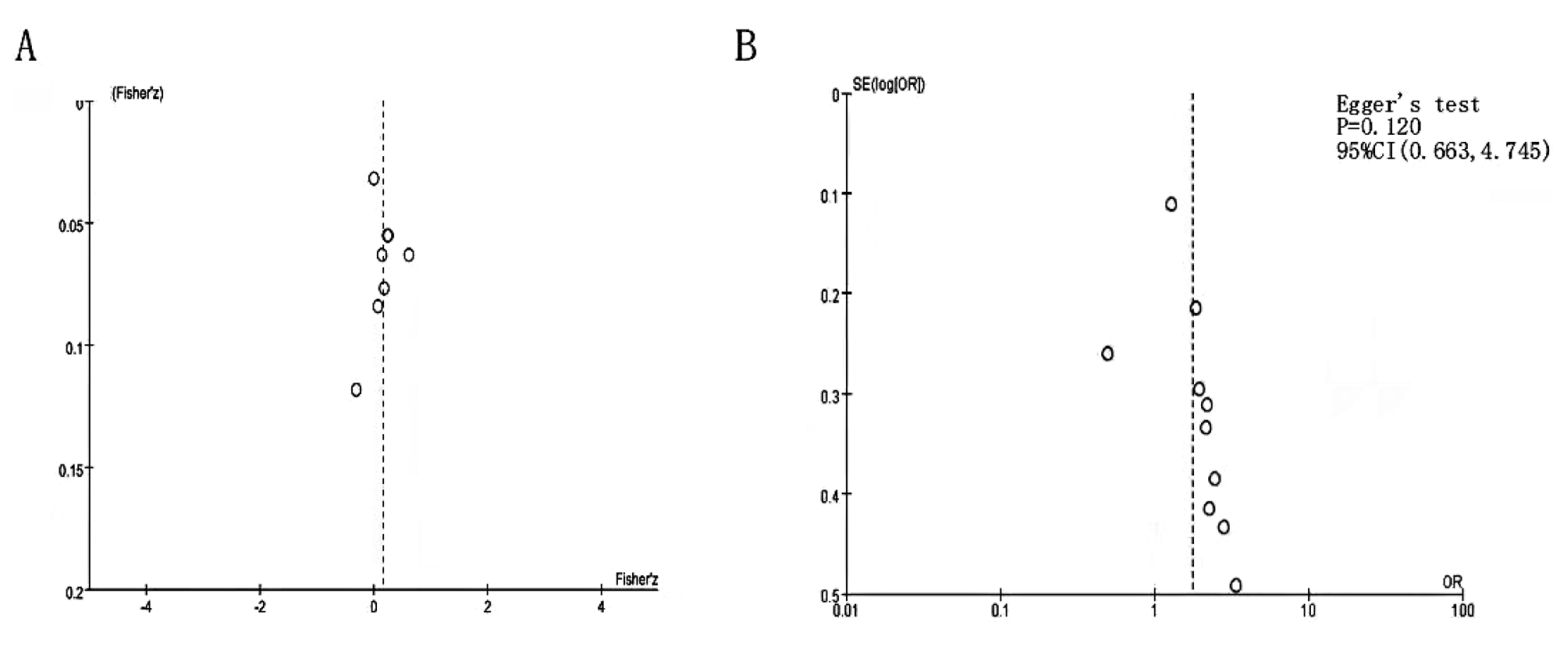
Funnel plot for the relationship between TSH and BMD (**A**); the relationship between TSH and osteoporosis (**B**)

Publication bias was assessed using Egger’s test. Taking effect as the publication bias of Pearson correlation coefficient, it can be seen that the overall sample size included in the study was large and bilateral symmetric, and the publication bias with effect size as OR value is found to be P = 0.120, 95%CI (-0.663, 4.745). Therefore, no single article appears to influence the combined results (Fig. 7).

## Discussion

Overt hypothyroidism is known to lower bone turnover by reducing both osteoclastic bone resorption and osteoblastic activity. These changes in bone metabolism would result in an increase in bone mineralization [28]. It is reported that the lifetime risk of osteoporosis in women is 40%-50% [29]. There were about 30% postmenopausal women who had osteoporosis in China [30]. However the relationship between serum TSH and BMD in postmenopausal women was still controversial, this study conducted meta-analysis and found that postmenopausal women’ s serum TSH was positively correlated with BMD, r = 0.158. The risk of osteoporosis in postmenopausal women with low-level TSH was 1.76 times of those with high-level TSH, 95% CI (1.27, 2.45). The dose-response relationship showed that when TSH was above 2.5mIu/L, the incidence of osteoporosis tended to be decreased. These results provided a theoretical basis for the prevention and treatment of osteoporosis in postmenopausal women.

**Fig. 8:**
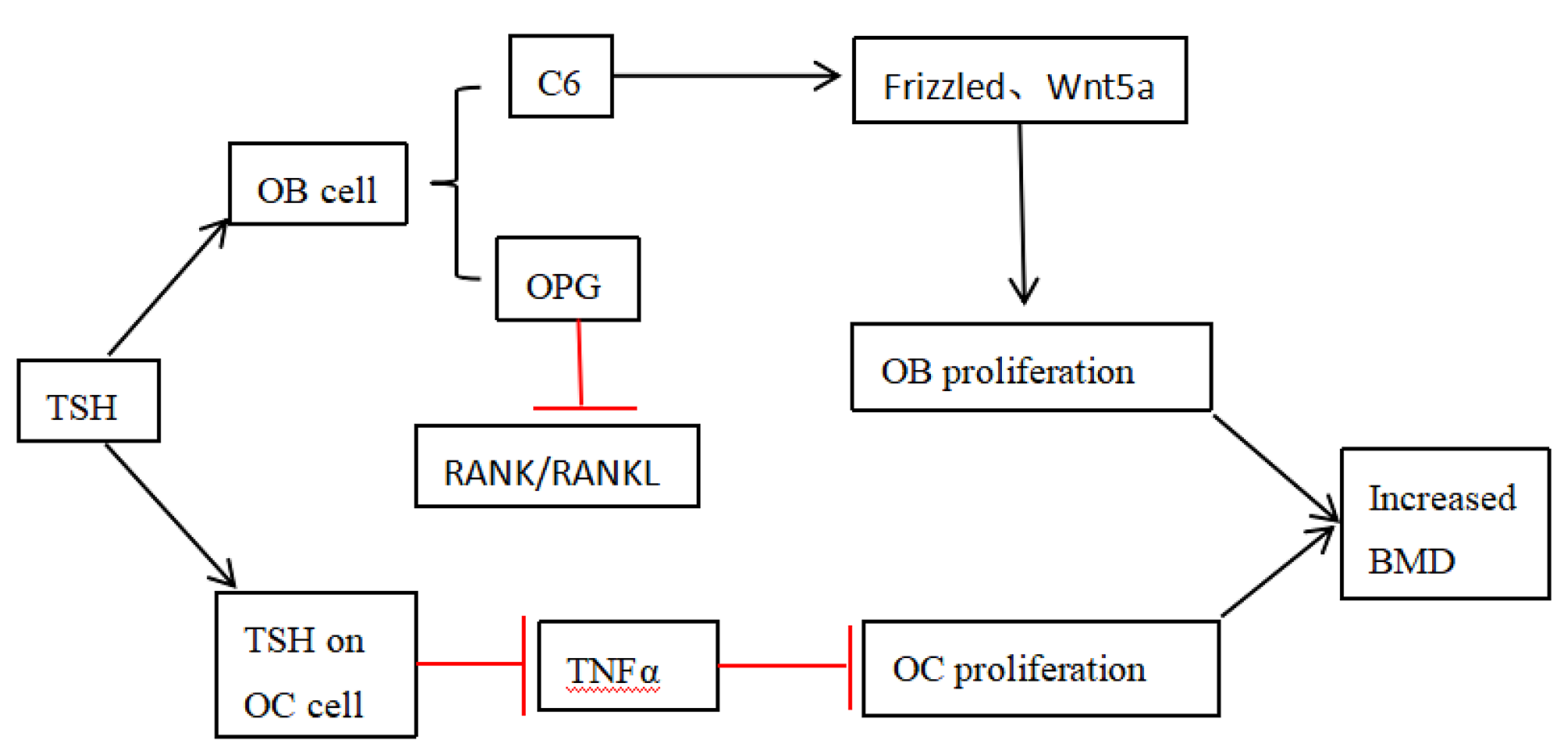
The mechanism of TSH involved in bone metabolism

Studies have demonstrated that serum TSH acts independently from thyroid hormone in bone metabolism. Wang Jiadan et al. [22]. found that the fluctuation of TSH level in the reference range in postmenopausal women with normal thyroid function may have a certain impact on the BMD of femoral neck, total hip and ward triangle. In addition, the TSH level may be an independent influencing factor of BMD in femoral neck and ward triangle, and those with low TSH level have a higher risk of osteopenia. TSH gets involved in bone turnover mainly through the following aspects: Firstly, TSH can promote osteoblast to secrete OPG, which can competitively inhibit the binding of RANK and RANKL, thus curbing the differentiation of osteoclast precursor cells into osteoclast. Secondly, TSH can activate protein kinase C6 in osteoblast to up-regulate frizzled and Wnt5a in non-canonical pathways, and induce osteoblast differentiation [31], which leaded to increasing level of BMD. This is consistent with the changing trend of TSH and BMD in the study of Wang Xiaodong [32]. The dose-response relationship showed that if the TSH level was below 2.5mIu/L, the risk of osteoporosis gradually increased, but TSH was up to 2.5mIu/L, it decreases with the increase of TSH level. BMD of the high-level TSH group was higher than that of the low-level TSH group, comparing with the BMD of the medium-level TSH group. It demonstrated that TSH was elevated when the BMD increased. Thirdly, TSH inhibits TNF-α and the proliferation and differentiation of osteoclast by binding to TSHR expressed on the surface of osteoclast [31]. In addition, TSH inhibits the binding of RANK and RANKL by directly inhibiting RANKL [33], thereby containing osteoclast precursor cells differentiate into osteoclast and curbing the formation of osteoclast [34] (Fig. 8), and reducing bone resorption.

From the Subgroup analysis it was found that the low-level TSH population from the community had a higher rate of osteoporosis than the patients from hospitals. Whether the anti-osteoporosis drugs were taken or not usually had no influence on the heterogeneity. According to subgroup analysis results targeting the different body part of BMD measurement and whether the subjects suffer from diabetes, we found that the relationship between TSH and BMD constant.

This study has a few limitations. First is that the articles included in this study are from cross-sectional survey and only conducted one measurement of TSH and BMD. Second, the value of BMD is not continuously measured as TSH changes. It will be more convincing that TSH and BMD be measured at a certain interval with multiple measurements. However, the concerned studies were tested for publication bias, and the results illustrated that there was no bias. Sensitivity analysis indicated that Wang Yi’s article [8] was highly sensitive, so it was excluded in the analysis. In future, the controlled trial should be conducted to clarify the relationship between TSH and BMD in postmenopausal women. At the same time, in vitro and in vivo experiments ought to be carried out to further explore how TSH promotes the secretion of OPG by osteoblast, and the specific signaling pathways and molecules that play a role in inhibiting the proliferation and differentiation of osteoclast.

## Conclusions

In summary, the serum TSH in the normal range of postmenopausal women was positively correlated with BMD, and high level (> 2.5mIu/L) within the normal range is helpful to decrease the risk of osteoporosis in postmenopausal women.

## Electronic supplementary material

Below is the link to the electronic supplementary material.

Additional File 1: PRISMA 2020 Checklist

Additional File 2: Dose response relationship code

Additional File 3: Quality evaluation of included articles methodology

Additional File 4: The OR value of different TSH levels and osteoporosis

Additional File 5: Results of sensitivity analysis based on the Pearson coefficient

Additional File 6: Dose response relationship data

## Data Availability

Data will be available upon request from the corresponding author.

## Abbreviations

BMD: bone mineral density
OP: osteoporosis
TSH: thyroid-stimulating hormone
OR: Odds ratio
