# Supplementary material for: A systematic review of the relationship between normal range of serum thyroid-stimulating hormone and bone mineral density in the postmenopausal women
Source: BMC Womens Health. 2023 Jul 5;23:358. doi: 10.1186/s12905-023-02488-9 (PMC10320894; doi:10.1186/s12905-023-02488-9)
Supplement: Supplementary file 2 — Additional File 2: Dose response relationship code [file 12905_2023_2488_MOESM2_ESM.doc]

**A Systematic Review of the Relationship between Normal Range of serum thyroid-stimulating hormone and bone mineral density in the postmenopausal women**

Xiaoli Zhu,^1^ Man Li,^1^  Shugang Li,^2^ Yifei Hu^2^

^1^:These two authors contribute equally to this work. Department of Public Health, School of Medicine, Capital Medical University, Beijing, China

^2^:Corresponding authors at:Department of Child, Adolescent Health and Maternal Care, School of Public Health, Capital Medical University, No. 10 You’ anmenwai Xitoutiao, Fengtai District, Beijing 100069, China.

E-mail addresses: [lishugang@ccmu.edu.cn(Shugang](mailto:lishugang@ccmu.edu.cn(Shugang) Li), [huyifei@yahoo.com(Yifei](mailto:huyifei@yahoo.com(Yifei) Hu).

Additional files2:Dose response relationship code

bysort id:gen tshc=tsh-tsh[1]

. gen logor =ln(or)

. gen loglb=ln(lb)

. gen logub=ln(ub)

. gen selogor=(logub-loglb)/(2*invnorm(.975))

. mkspline tshcs=tshc,nk(3) cubic

. mkspline tshs=tsh,nk(3) cubic

. mvmeta_make glst logor tshcs1 tshcs2,cov (n case) se (selogor) pfirst (id studyt) saving (ssest_spline

> ) replace by (id) names (b V)

Using coefficients: tshcs1 tshcs2

-> id==1

Fixed-effects dose-response model Number of studies = 1

Generalized least-squares regression Number of obs = 2

Goodness-of-fit chi2(0) = . Model chi2(2) = 7.11

Prob > chi2 = . Prob > chi2 = 0.0285

------------------------------------------------------------------------------

logor | Coef. Std. Err. z P>|z| [95% Conf. Interval]

-------------+----------------------------------------------------------------

tshcs1 | -.5689367 .2283071 -2.49 0.013 -1.01641 -.1214629

tshcs2 | .3643981 .1396125 2.61 0.009 .0907627 .6380335

------------------------------------------------------------------------------

Note:logor:the log of the OR value coef:coefficient Std.Err: The standard error of the regression coefficient z: z value P>|z|：the probability that P is greater than z

-> id==2

Fixed-effects dose-response model Number of studies = 1

Generalized least-squares regression Number of obs = 2

Goodness-of-fit chi2(0) = . Model chi2(2) = 5.02

Prob > chi2 = . Prob > chi2 = 0.0813

------------------------------------------------------------------------------

logor | Coef. Std. Err. z P>|z| [95% Conf. Interval]

-------------+----------------------------------------------------------------

tshcs1 | -.1607528 .0718252 -2.24 0.025 -.3015277 -.019978

tshcs2 | .0924474 .0589445 1.57 0.117 -.0230817 .2079765

------------------------------------------------------------------------------

Note:logor:the log of the OR value coef:coefficient Std.Err: The standard error of the regression coefficient z: z value P>|z|：the probability that P is greater than z

-> id==3

Fixed-effects dose-response model Number of studies = 1

Generalized least-squares regression Number of obs = 2

Goodness-of-fit chi2(0) = . Model chi2(2) = 8.43

Prob > chi2 = . Prob > chi2 = 0.0147

------------------------------------------------------------------------------

logor | Coef. Std. Err. z P>|z| [95% Conf. Interval]

-------------+----------------------------------------------------------------

tshcs1 | -.210009 .0726407 -2.89 0.004 -.3523822 -.0676357

tshcs2 | -8.131297 7.964362 -1.02 0.307 -23.74116 7.478566

------------------------------------------------------------------------------

Note:logor:the log of the OR value coef:coefficient Std.Err: The standard error of the regression coefficient z: z value P>|z|：the probability that P is greater than z

-> id==4

Fixed-effects dose-response model Number of studies = 1

Generalized least-squares regression Number of obs = 3

Goodness-of-fit chi2(1) = 0.50 Model chi2(2) = 7.12

Prob > chi2 = 0.4813 Prob > chi2 = 0.0285

------------------------------------------------------------------------------

logor | Coef. Std. Err. z P>|z| [95% Conf. Interval]

-------------+----------------------------------------------------------------

tshcs1 | -.215559 .0852891 -2.53 0.011 -.3827226 -.0483954

tshcs2 | -.8632607 2.611298 -0.33 0.741 -5.98131 4.254789

------------------------------------------------------------------------------

Note:logor:the log of the OR value coef:coefficient Std.Err: The standard error of the regression coefficient z: z value P>|z|：the probability that P is greater than z

-> id==5

Fixed-effects dose-response model Number of studies = 1

Generalized least-squares regression Number of obs = 5

Goodness-of-fit chi2(3) = 0.47 Model chi2(2) = 7.11

Prob > chi2 = 0.9245 Prob > chi2 = 0.0286

------------------------------------------------------------------------------

logor | Coef. Std. Err. z P>|z| [95% Conf. Interval]

-------------+----------------------------------------------------------------

tshcs1 | -.2600489 .0987672 -2.63 0.008 -.4536291 -.0664687

tshcs2 | .2068407 1.096502 0.19 0.850 -1.942264 2.355945

------------------------------------------------------------------------------

Note:logor:the log of the OR value coef:coefficient Std.Err: The standard error of the regression coefficient z: z value P>|z|：the probability that P is greater than z

file ssest_spline.dta saved

. preserve

. use ssest_spline,clear

.

. mvmeta b V,mm i2

Note: using method mm (truncated)

Note: using variables btshcs1 btshcs2

Note: 5 observations on 2 variables

------------------------------------------------------------------------------

| Coef. Std. Err. z P>|z| [95% Conf. Interval]

-------------+----------------------------------------------------------------

btshcs1 | -.2233323 .0626101 -3.57 0.000 -.3460458 -.1006187

btshcs2 | .1370972 .065637 2.09 0.037 .008451 .2657433

------------------------------------------------------------------------------

Note:logor:the log of the OR value coef:coefficient Std.Err: The standard error of the regression coefficient z: z value P>|z|：the probability that P is greater than z

Estimated between-studies SDs and correlation matrix:

SD btshcs1 btshcs2

btshcs1 .10505648 1 -1

btshcs2 .12096804 -1 1

. testparm btshcs2

( 1) btshcs2 = 0

chi2( 1) = 4.36

Prob > chi2 = 0.0367

. capture estimates save mvmeta, replace

.

. restore

. estimates use mvmeta

. recast float tshs1

. recast float tshs2, force

tshs2: 14 values changed

. glst logor tshs*,se(se) cov(n case) pfirst(id studyt)

Fixed-effects dose-response model Number of studies = 5

Generalized least-squares regression Number of obs = 14

Goodness-of-fit chi2(12) = 26.92 Model chi2(2) = 8.84

Prob > chi2 = 0.0079 Prob > chi2 = 0.0120

------------------------------------------------------------------------------

logor | Coef. Std. Err. z P>|z| [95% Conf. Interval]

-------------+----------------------------------------------------------------

tshs1 | .176745 .0613262 2.88 0.004 .0565478 .2969422

tshs2 | -.2142816 .085909 -2.49 0.013 -.3826602 -.045903

------------------------------------------------------------------------------

Note:logor:the log of the OR value coef:coefficient Std.Err: The standard error of the regression coefficient z: z value P>|z|：the probability that P is greater than z

. recast double tshs1

. recast double tshs2

. predictnl logor_sp=_b[tshs1]*tshs1+_b[tshs2]*tshs2,ci(low up)

note: Confidence intervals calculated using Z critical values

.

. note: Confidence intervals calculated using Z critical values

. gen ors=exp(logor_sp)

. gen lbs=exp(low)

. gen ubs=exp(up)

. twoway (line ors lbs ubs tsh,sort lc(black black black) lp(l longdash longdash)), yscale(log) ytitle("

> Odds ratio") xtitle("Thyroid Stimulating Hormon") scheme (s1mono) xlabel(0(1)8) xmtick(0(0.5)8) ymtick

> (0(0.5)2) ylabel(1 1.5 2, format(%3.2fc) angle(horiz)) plotregion (style(none))
