# Supplementary material for: A systematic review of the relationship between normal range of serum thyroid-stimulating hormone and bone mineral density in the postmenopausal women
Source: BMC Womens Health. 2023 Jul 5;23:358. doi: 10.1186/s12905-023-02488-9 (PMC10320894; doi:10.1186/s12905-023-02488-9)
Supplement: Supplementary file 3 — Additional File 3: Quality evaluation of included articles methodology [file 12905_2023_2488_MOESM3_ESM.docx]

Additional file 3 Quality evaluation of included articles methodology

| Included in the study | Source of research objects | Inclusion and exclusion criteria | Research object time period | Study object continuity | Other conditions of research subjects | Reassess | Exclude reasons for analysis | Control measures for confounding factors | Lost data handling | Response and data collection integrity | Follow up | The literature class |
| --- | --- | --- | --- | --- | --- | --- | --- | --- | --- | --- | --- | --- |
| Duk Jae kim 2006 | 1 | 1 | 1 | 1 | 0 | 1 | 1 | 0 | 2 | 2 | 2 | medium |
| Martha.Savaria Morris 2007 | 1 | 1 | 2 | 1 | 0 | 1 | 2 | 1 | 2 | 2 | 2 | medium |
| Gherardo Mazziotti 2010 | 1 | 1 | 1 | 1 | 0 | 1 | 2 | 0 | 2 | 2 | 0 | medium |
| Avi Leader 2014 | 1 | 1 | 1 | 1 | 0 | 1 | 2 | 0 | 2 | 2 | 1 | medium |
| H.-M.Noh 2015 | 1 | 1 | 1 | 1 | 0 | 1 | 1 | 1 | 2 | 2 | 2 | medium |
| Yin Fei（Chinese）2016 | 1 | 1 | 1 | 1 | 0 | 1 | 2 | 0 | 2 | 2 | 2 | medium |
| Berrin Acar 2016 | 1 | 1 | 1 | 1 | 0 | 1 | 1 | 0 | 2 | 2 | 2 | medium |
| Bo Ding 2016 | 1 | 1 | 1 | 1 | 0 | 1 | 2 | 0 | 2 | 2 | 2 | medium |
| SuJinLee 2016 | 1 | 1 | 1 | 1 | 0 | 1 | 1 | 0 | 2 | 2 | 2 | medium |
| Lin Mei (Chinese) 2016 | 1 | 1 | 1 | 1 | 0 | 1 | 2 | 2 | 2 | 2 | 2 | medium |
| Wang Jiadan (Chinese) 2017 | 1 | 1 | 1 | 1 | 0 | 1 | 2 | 0 | 2 | 2 | 2 | medium |
| Wang Yi (Chinese）2018 | 1 | 1 | 1 | 1 | 0 | 1 | 1 | 0 | 2 | 2 | 2 | medium |
| Niu Fengxiu (Chinese) 2018 | 1 | 1 | 1 | 1 | 0 | 1 | 2 | 2 | 2 | 2 | 2 | medium |
| Qin Liping (Chinese) 2018 | 1 | 1 | 1 | 1 | 0 | 1 | 2 | 2 | 2 | 2 | 2 | medium |
| Gao Saisai (Chinese) 2019 | 1 | 1 | 1 | 1 | 0 | 1 | 2 | 0 | 2 | 2 | 2 | medium |
| Zhang Lihong (Chinese) 2019 | 1 | 1 | 1 | 1 | 0 | 1 | 2 | 0 | 2 | 2 | 2 | medium |
| Chen Qingling (Chinese) 2019 | 1 | 1 | 1 | 1 | 2 | 1 | 2 | 0 | 2 | 2 | 1 | medium |
| Cui Xinjie (Chinese) 2020 | 1 | 1 | 1 | 1 | 2 | 1 | 2 | 0 | 2 | 2 | 2 | medium |

Note: 1 Yes 2 No 0 Unclear
