# Supplementary material for: A systematic review of the relationship between normal range of serum thyroid-stimulating hormone and bone mineral density in the postmenopausal women
Source: BMC Womens Health. 2023 Jul 5;23:358. doi: 10.1186/s12905-023-02488-9 (PMC10320894; doi:10.1186/s12905-023-02488-9)
Supplement: Supplementary file 4 — Additional File 4: The OR value of different TSH levels and osteoporosis [file 12905_2023_2488_MOESM4_ESM.docx]

Additional file 4 The OR value of different TSH levels and osteoporosis

| Included in the study | Average TSH (mIU/L) | OR | 95%CI |
| --- | --- | --- | --- |
| Chen Qingling 2019 | 2.665 | 1.00 | (1，1) |
|  | 1.1 | 2.63 | (1.23，5.592) |
|  | 7.17 | 1.22 | (0.88，1.689) |
| Avi Leader 2014 | 2.3 | 1.00 | (1，1) |
|  | 0.975 | 1.28 | (1.03，1.59) |
|  | 3.6 | 1.12 | (0.82，1.53) |
| Su JinLee 2016 | 3.72 | 1.00 | (1，1) |
|  | 0.765 | 1.86 | (1.22，2.83) |
|  | 1.555 | 1.30 | (0.86，1.97) |
| H._M.Noh 2015 | 4.715 | 1.00 | (1，1) |
|  | 0.96 | 2.169 | (1.128，4.171) |
|  | 1.99 | 2.10 | (1.12，3.921) |
|  | 2.9 | 1.42 | (0.73，2.759) |
| Duk Jae Kim 2016 | 3.9 | 1.00 | (1，1) |
|  | 1 | 2.66 | (0.91，7.83) |
|  | 0.8 | 2.19 | (1.19，4.04) |
|  | 1.35 | 1.69 | (0.89，3.99) |
|  | 1.75 | 1.75 | (0.92，3.35) |
|  | 2.35 | 1.52 | (0.81，2.85) |
