# Supplementary material for: A systematic review of the relationship between normal range of serum thyroid-stimulating hormone and bone mineral density in the postmenopausal women
Source: BMC Womens Health. 2023 Jul 5;23:358. doi: 10.1186/s12905-023-02488-9 (PMC10320894; doi:10.1186/s12905-023-02488-9)
Supplement: Supplementary file 5 — Additional File 5: Results of sensitivity analysis based on the Pearson coefficient [file 12905_2023_2488_MOESM5_ESM.docx]

Additional file 5 Results of sensitivity analysis based on the Pearson coefficient

| Exclude included studies | Sample size | Fisher’ Z | 95%CI | I^2^ | P |
| --- | --- | --- | --- | --- | --- |
| Lin JD 2011 | 974 | 0.18 | (0.02,0.35) | 91% | 0.03 |
| Yin Fei 2016 | 135 | 0.17 | (-0.01,0.34) | 94% | 0.06 |
| Lin Mei 2016 | 166 | 0.16 | (-0.02,0.33) | 94% | 0.09 |
| Wang Jiadan 2017 | 234 | 0.16 | (-0.02,0.34) | 94% | 0.08 |
| Wang Yi 2018 | 110 | 0.19 | (0.01,0.38) | 94% | 0.04 |
| Niu Fengxiu 2018 | 308 | 0.14 | (-0.04,0.33) | 94% | 0.12 |
| Gao Saisai 2019 | 267 | 0.10 | (-0.01,0.21) | 83% | 0.08 |
| Zhang Lihong 2019 | 72 | 0.21 | (0.06,0.37) | 93% | 0.008 |
| Cui Xinjie 2020 | 307 | 0.15 | (-0.04,0.33) | 94% | 0.11 |
