# Supplementary material for: A systematic review of the relationship between normal range of serum thyroid-stimulating hormone and bone mineral density in the postmenopausal women
Source: BMC Womens Health. 2023 Jul 5;23:358. doi: 10.1186/s12905-023-02488-9 (PMC10320894; doi:10.1186/s12905-023-02488-9)
Supplement: Supplementary file 6 — Additional File 6: Dose response relationship data [file 12905_2023_2488_MOESM6_ESM.doc]

**A Systematic Review of the Relationship between Normal Range of serum thyroid-stimulating hormone and bone mineral density in the postmenopausal women**

Xiaoli Zhu,^1^ Man Li,^1^  Shugang Li,^2^ Yifei Hu^2^

^1^:These two authors contribute equally to this work. Department of Public Health, School of Medicine, Capital Medical University, Beijing, China

^2^:Corresponding authors at:Department of Child, Adolescent Health and Maternal Care, School of Public Health, Capital Medical University, No. 10 You’ anmenwai Xitoutiao, Fengtai District, Beijing 100069, China.

E-mail addresses: [lishugang@ccmu.edu.cn(Shugang](mailto:lishugang@ccmu.edu.cn(Shugang) Li), [huyifei@yahoo.com(Yifei](mailto:huyifei@yahoo.com(Yifei) Hu).

Additional files6: Dose response relationship data

| Id | interval | tsh | case | control | n | or | lb | ub | study |
| --- | --- | --- | --- | --- | --- | --- | --- | --- | --- |
| 1 | 0.55-4.78 | 2.665 | 148.00 | 2965.00 | 3113 | 1.00 | 1.00 | 1 | 1 |
| 1 | ＜0.55 mIU/L | 1.1 | 8.00 | 63.00 | 71 | 2.63 | 1.23 | 5.592 | 1 |
| 1 | ﹥4.78 | 7.17 | 51.00 | 838.00 | 889 | 1.22 | 0.88 | 1.689 | 1 |
| 2 | 1.7-2.9 | 2.3 | 141.00 | 3626.00 | 3767 | 1.00 | 1.00 | 1 | 1 |
| 2 | 0.35–1.6mIU/L | 0.975 | 211.00 | 4058.00 | 4269 | 1.28 | 1.03 | 1.59 | 1 |
| 2 | 3-4.2 | 3.6 | 60.00 | 1325.00 | 1385 | 1.12 | 0.82 | 1.53 | 1 |
| 3 | 1.94-5.50 | 3.72 | 105.00 | 115.00 | 220 | 1.00 | 1.00 | 1 | 1 |
| 3 | 0.36-1.17 | 0.765 | 116.00 | 111.00 | 227 | 1.86 | 1.22 | 2.83 | 1 |
| 3 | 1.18-1.93 | 1.555 | 114.00 | 113.00 | 227 | 1.30 | 0.86 | 1.97 | 1 |
| 4 | 3.43-6.00 | 4.715 | 21.00 | 104.00 | 125 | 1.00 | 1.00 | 1 | 1 |
| 4 | 0.32-1.60 | 0.96 | 35.00 | 81.00 | 116 | 2.16 | 1.14 | 4.077 | 1 |
| 4 | 1.61-2.37 | 1.99 | 37.00 | 92.00 | 129 | 2.10 | 1.12 | 3.921 | 1 |
| 4 | 2.38-3.42 | 2.9 | 25.00 | 99.00 | 124 | 1.42 | 0.73 | 2.759 | 1 |
| 5 | 2.8-5.00 | 3.9 | 20.00 | 167.00 | 187 | 1.00 | 1.00 | 1 | 1 |
| 5 | ＜0.5 | 1 | 6.00 | 19.00 | 25 | 2.66 | 0.91 | 7.83 | 1 |
| 5 | 0·5–1·1 mU/L | 0.8 | 45.00 | 153.00 | 198 | 2.19 | 1.19 | 4.04 | 1 |
| 5 | 1·2–1·5 mU/L | 1.35 | 30.00 | 153.00 | 183 | 1.69 | 0.89 | 3.99 | 1 |
| 5 | 1·6–1·9 mU/L | 1.75 | 28.00 | 135.00 | 163 | 1.75 | 0.92 | 3.35 | 1 |
| 5 | 2·0–2·7 mU/L | 2.35 | 31.00 | 172.00 | 203 | 1.52 | 0.81 | 2.85 | 1 |

Note: Id:The serial number interval：intervention dose tsh: thyrotropin

case:Case group control:The control group n:Sample size

or:Effect size of each exposure group in the study

lb: The lower limit of the effect size of each exposure group

ub:The upper limit of the effect size of each exposure group

| study | OR | LCI | UCI | source | drug |
| --- | --- | --- | --- | --- | --- |
| Duk Jae Kim2006 | 2.19 | 1.19 | 4.04 | community | no anti-op |
| Martha Savaria Morris2007 | 3.4 | 1.3 | 9.2 | community | take anti-op |
| Gherardo Mazziotti2010 | 2.8 | 1.2 | 6.79 | hospital | no anti-op |
| Avi Leader2014 | 1.28 | 1.03 | 1.59 | community | take anti-op |
| H._M.Noh2015 | 2.169 | 1.128 | 4.171 | community | take anti-op |
| Berrin Acar2016 | 0.5 | 0.3 | 0.8 | hospital | no anti-op |
| Bo Ding2016 | 1.96 | 1.1 | 3.493 | hospital | no anti-op |
| Qinliping2018 | 2.278 | 1.011 | 5.132 | community | no anti-op |
| Su jin Lee2016 | 1.86 | 1.22 | 2.83 | community | no anti-op |
| Chenqingling2019 | 2.626 | 1.233 | 5.592 | community | no anti-op |

Note: OR:Odds ratio LCI: lower limit of confidence interval

UCI: upper limit of confidence interval  source:Source object

drug：medication use
